# Seed Dispersers, Seed Predators, and Browsers Act Synergistically as Biotic Filters in a Mosaic Landscape

**DOI:** 10.1371/journal.pone.0107385

**Published:** 2014-09-18

**Authors:** Regino Zamora, Luis Matías

**Affiliations:** 1 Grupo de Ecología Terrestre, Departamento de Ecología, Facultad de Ciencias, Universidad de Granada, Granada, Spain; 2 Biological and Environmental Sciences, School of Natural Sciences, University of Stirling, Stirling, United Kingdom; Key Laboratory of Tropical Forest Ecology, Xishuangbanna Tropical Botanical Garden, Chinese Academy of Sciences, China

## Abstract

In this study, we analize the functional influence of animals on the plants they interact with in a mediterranean mountain. We hypothesise that seed dispersers, seed predators, and browsers can act as biotic filters for plant communities. We analyse the combined effects of mutualistic (seed dispersal) and antagonistic (seed predation, herbivory) animal interactions in a mosaic landscape of Mediterranean mountains, basing our results on observational and experimental field. Most of the dispersed seeds came from tree species, whereas the population of saplings was composed predominantly of zoochorous shrub species. Seed predators preferentially consumed seeds from tree species, whereas seeds from the dominant fleshy-fruited shrubs had a higher probability of escaping these predators. The same pattern was repeated among the different landscape units by browsers, since they browsed selectively and far more intensely on tree-species saplings than on the surrounding shrubs. In synthesis, our work identifies the major biotic processes that appear to be favoring a community dominated by shrubs versus trees because seed dispersers, predators, and herbivores together favored shrub dispersal and establishment versus trees.

## Introduction

The demographic consequences of plant-animal interactions have constituted a key topic for ecological studies in recent decades [1]. It has been commonly accepted that mutualistic and antagonistic interactions have opposite consequences and therefore induce different responses in plant demography [2]–[4]. By selective dispersal of the seeds contained in the fruits consumed, mutualistic animal species are able to alter the relative composition and abundance of plant communities, favouring species with which they interact most frequently (*sensu* [5]). Likewise, antagonistic animal species (seed predators and browsers) can have similar effects on plant populations, selectively consuming more palatable species or those with lower defences while avoiding less palatable species, thereby filtering community composition and granting a competitive edge to species with physical or chemical defences [6]–[9]. In this context, the relative abundance of the different species in a given plant community would be viewed as the result of the selection by mutualistic and/or antagonistic biotic filters over the regional species pool [10]–[12].

In Mediterranean ecosystems, contrary to temperate and tropical ones, summer drought has been traditionally considered the major limiting factor for plant recruitment among a wide diversity of habitats such as lowland forests [13], [14], mountain forests [15], [16], shrublands [17], [18], and semiarid ecosystems [19]. However, recent evidence such as the high proportion of zoochorous species or the role of seed predators and browsers point to the under-appreciated importance of biotic factors on the dynamics of woody-species communities [4], [15], [20], [21]. Thus, the identification of selection patterns, and their spatio-temporal consistency, would provide a fuller understanding of the consequences of plant-animal interactions as biotic filters for the regeneration of the woody community across different landscape units, representing a gradient between “natural” or “wild” ecosystems on one hand to intensively managed systems on the other [22]. In fact, a growing body of evidence supports the rearrangement of native biotic assemblages subjected to human-induced disturbances where some species may come to dominate ecological communities while others are detrimentally impacted [23]. This community reorganization processes are in fact occurring in the tropics [24], but the extent to which this phenomenon applies to other terrestrial biomes remains largely unknown. More importantly, we hardly know the relative importance and spatio-temporal consistency of biotic and abiotic mechanisms causing species replacements and community reorganization.

In this study, we analyse the combined effects of seed dispersers, seed predators, and browsers in a mosaic landscape composed of patches of native forest and degraded habitats representative of Mediterranean mountains. We sampled landscape mosaics shaped by human disturbance, all chosen to represent gradients of habitat and fruit resource availability. For this we conducted simultaneous field experiments on three plant-animal interactions gathered in the same area and at the same time. In previous studies [20], [25], [26], [27], we analysed the magnitude and relative importance of abiotic and biotic factors on recruitment limitation (by comparing seed vs. establishment limitation) for the woody community in three Mediterranean sites differing in type of degradation: native forest (used as control), reforestation stands, and post-fire shrubland. We found that, overall, recruitment in the woody community was severely limited by both seed dispersal and establishment. However, tree species were more establishment-limited than shrubs. Trees and shrubs represent the two dominant woody vegetation types in terrestrial ecosystems, and our analyses show that their formation will depend on the impact of the abiotic and biotic filters on vegetation dynamics on a specific community.

In this context, taking into account that recruitment in this Mediterranean woody community is both severely seed and establishment limited due to both abiotic and biotic factors [20], [25], [27], we questioned the role that seed dispersers, seed predators, and browsers play as ecological filters for the woody-plant community across different landscape units. More specifically, we asked: 1) Do mutualistic and antagonistic plant-animal interactions have similar effects on the different functional groups of a woody community? 2) What are the demographic consequences of the mutualistic and antagonistic selective filters in the recruitment bank across a degraded mosaic landscape? 3) To what extent mutualistic and antagonistic animals does create a favourable selective environment for the same functional types of plants, irrespective of habitat? With this approach we seek to determine the combined role of seed dispersers, seed predators and browsers as biotic filters for the regeneration of the woody community across different landscape units, representing a gradient of human management.

## Materials and Methods

The present study was conducted at Sierra Nevada National Park, within an area located between 1600 and 1900 m a.s.l. (37°05′N, 3°28′W, SE Spain). All necessary permits for the field studies described herein (which did not involve endangered or protected species) were obtained thanks to Javier Sanchez, Director of Sierra Nevada Nacional Park. The climate in this area is continental Mediterranean, with annual rainfall of 811 mm (mean values for the 1990–2010 period) concentrated in autumn and spring. Winters are cold whereas summers are hot and dry, mean temperatures of the coldest (January) and hottest months (July) are 3.6 and 21.5°C, respectively. The frugivorous guild in this area is composed of small birds (Robin, *Erithacus rubecula*, and Blackcap, *Sylvia atricapilla*; 12–20 g) and medium-sized birds (thrushes, *Turdus* spp.; 60–120 g). Most of these species are sedentary in the study area, except the Redwing (*T. iliacus*) and the Ring-ouzel (*T. torquatus*), which are long-distance migrant birds. All of these species are omnivorous, have a frugivorous diet during autumn–early winter, and are legitimate seed dispersers of many fruit-bearing plants in Mediterranean mountains [20], [28]. Previous studies in the same study area [29], [30] have reported by means of field observations and trapping that the main post-dispersal seed predators are woodmouse (*Apodemus sylvaticus*) and wild boar (*Sus scrofa*), while the browser guild is composed of ungulates, wild (Spanish ibex, *Capra pyrenaica*) as well as domestic (sheep, goats, and cattle). This assemblage have a full representation of the different types of animals interacting with plants (dispersers, seed predators, browsers), allowing us to examine the relative importance of the biotic filters on the configuration of the plant community from seed dispersal to late recruitment.

As a result of a long history of land use, the study area is a mosaic landscape composed of patches of three main habitats: native forest, dominated by *Pinus sylvestris* mixed with other trees such as *Acer opalus*, *Sorbus aria* or *Taxus baccata*, as well as a dense shrubby understorey formed by fleshy-fruited shrubs such as *Crataegus monogyna*, *Berberis vulgaris*, *Juniperus communis* or *Prunus ramburii*, and dry-fruited shrubs such as *Salvia lavandulifolia*, *Cytisus scoparius* or *Ononis aragonensis*, among others. The next two habitats were reforestation stands, planted in the 1950s and dominated by *P. sylvestris*, where we distinguished two different management levels: (2) dense stands (unmanaged, ca. 1040 trees ha^−1^) and (3) cleared stands (with a pine density reduced by about 50%) and (4) post-fire shrublands, dominated by the aforementioned fleshy- and dry-fruited shrubs. The first habitat represents a low degradation habitat, whereas the reforestation stands and the shrubland constitute two contrasting types of, novel, human-created habitats [22].

Each landscape unit was represented by three plots of similar size (mean = 0.53 ha±0.06, average distance between the three plots of the same landscape unit = 688 m), giving a total of 12 study plots (a map showing the spatial distribution of the study plots can be found in [25]). In each landscape unit, we determined the diversity and abundance of tree and shrub adults (i.e. reproductive individuals), by counting all the trees and shrubs in each plot. We also collected the following data from each plot:

1. *Seed rain* was quantified by the use of seed traps from October 2003 to April 2005 (two complete dispersal seasons). Two paired seed traps (0.04 m^2^ surface) were located at 15 fixed points in each plot. Traps were protected against post-dispersal seed predation by a wire mesh of 1-cm grid size. Contents were collected every two months, and all seeds were counted and identified to the species level. From fleshy-fruited species, only bare seeds (those with fruits digested by animals and thus effectively dispersed) were taken into account for analyses, while all seeds were counted for anemochorous and autochorous species (see [25] for a full description).
2. *Post-dispersal seed predation* was tested by recording the removal of seeds of three tree species (*P. sylvestris*, *A. opalus*, and *S. aria*) as well as two fleshy-fruited shrub species (*C. monogyna* and *B. vulgaris*) exposed simultaneously to predators in the field. At the same points where seed traps were located, two squares (20×20 cm) of plastic mesh were pegged to the soil and two seeds of each species were glued in a random position with a low-odour thermoplastic adhesive to them; the squares were 2 m apart. The seeds were exposed to predators in March 2004 and 2005 (after the natural seed-dispersal period, but before seedling emergence) and monitored after 30 days, recording the proportion of seeds consumed by predators (see [26] for a full description).
3. *Sapling abundance and diversity* was monitored using two 1-m^2^ quadrats located at the same points where seed dispersal and predation were estimated. The identity and number of non-reproductive saplings older than two years was recorded in late spring 2004 and 2005 (May-June; see [25] for a full description).
4. *Damage by browsers* was quantified in established saplings of tree species (up to 2 m high) and in shrubs by the determination of browsing intensity in September 2008. This was calculated as the accumulated proportion of apical shoots consumed by ungulates (mainly domestic and wild goats, *Capra aegagrus* and *C. hispanica*, respectively) during the last four years with respect to the total number of buds by the identification of the scars by shoot consumption [31], [32]. All buds were examined for herbivory in small saplings or shrubs (<1,5 m in heigh) and, due to their large size, 100 randomly selected buds in large ones (>1,5 m in height) noting the proportion of damaged buds. In each plot, we measured 20 randomly selected individuals from each of the most abundant tree and shrubby species (*P. sylvestris*, *A. opalus*, *S. aria*, *Q. ilex*, *C. monogyna*, *B. vulgaris, P. ramburii, Rosa canina, Juniperus communis, Adenocarpus decorticans, S. lavandulifolia*, *C. scoparius* and *O. aragonensis).*

Since we focus on the effect of mutualistic and antagonistic interactions at the community level, and given that the woody community includes a great number of species with different architecture and dispersal type (Table 1), all species were functionally grouped based on growth habit and dispersal type. As zoochorous trees were absent from the degraded habitats (Table 2), we included all tree species within the same group, thus clasifying the community into *trees*, *fleshy-fruited shrubs*, and *dry-fruited shrubs*.

**Table 1:** Species included within the different functional groups according to their growth architecture and dispersal type and their density, relative abundance and number of dispersed seeds in the study area.

Species	Dispersaltype	Density(ind. ha^−1^)	Relativeabundance (%)	Seed dispersal(seeds ha^−1^)
Trees				
* Acer opalus* Boiss.	Anemochorous	3.0	0.12	75.7
* Pinus nigra* Arnold	Anemochorous	42.6	1.70	33.9
* Pinus sylvestris* L.	Anemochorous	320.8	12.83	589.4
* Sorbus aria* (L.) Crantz	Zoochorous	0.4	0.02	0.4
* Taxus baccata* L.	Zoochorous	1.2	0.19	21.4
**Total trees**		**368**	**14.85**	**720.8**
Fleshy-fruited shrubs				
* Amelanchier ovalis* Medik.	Zoochorous	1.2	0.05	1.3
* Berberis vulgaris* L.	Zoochorous	46.7	1.87	79.8
* Cotoneaster granatensis* Boiss.	Zoochorous	1.7	0.07	0.4
* Crataegus monogyna* Jacq.	Zoochorous	103.3	4.13	90.6
* Juniperus communis* L.	Zoochorous	20.0	0.80	36.3
* Juniperus sabina* L.	Zoochorous	5.0	0.20	0.6
* Lonicera arborea* Boiss.	Zoochorous	5.9	0.24	13.1
* Lonicera xylosteum* L.	Zoochorous	58.3	2.33	6.25
* Prunus ramburii* Boiss.	Zoochorous	216.7	8.67	12.6
* Rosa canina* L.	Zoochorous	21.7	0.87	29.3*
* Rosa pimpinellifolia* L.	Zoochorous	58.3	2.33	-
* Rosa sicula* Tratt.	Zoochorous	41.7	1.67	-
* Rosa stylosa* Desv.	Zoochorous	13.3	0.53	-
* Rubus ulmifolius* Schott.	Zoochorous	3.3	0.13	8.4
**Total FFS**		**597.1**	**23.88**	**278.6**
Dry-fruited shrubs				
* Adenocarpus decorticans* Boiss.	Autochorous	3.3	0.13	0.5
* Astragalus granatensis* Lam.	Autochorous	26.7	1.07	-
* Cytisus scoparius* (L.) Link	Autochorous	6.7	0.27	0.4
* Erinacea anthyllis* Link	Autochorous	145.0	5.80	-
* Genista cinerea* (Vill.) DC.	Autochorous	8.3	0.33	-
* Ononis aragonensis* Asso	Autochorous	286.7	11.47	4.4
* Salvia lavandulifolia* Vahl.	Autochorous	1055.0	42.20	42.4
**Total DFS**		**1531.7**	**61.26**	**47.7**

All plots are pooled together.

*All seeds from the different species of the Genus *Rosa* are included together due to the difficulties to distinguish them.

**Table 2:** Density fo individuals (in individuals ha^−1^) by functional groups (AT: anemochorous trees; ZT: zoochorous tress; FFS: fleshy-fruited shrubs; DFS: dry-fruited shrubs) on the different study plots.

Habitat	Plot	AT	ZT	FFS	DFS
Native	1	104	97	617	32
	2	91	12	310	6
	3	87	4	123	19
Cleared	1	675	0	105	0
	2	454	0	63	25
	3	432	0	171	77
Dense	1	858	0	10	0
	2	1128	0	20	31
	3	1138	0	73	68
Shrubland	1	21	0	1172	2370
	2	21	0	1607	1293
	3	66	0	647	3117

### Data analysis

Differences in seed rain, predation rate and in browsing damage among habitats and functional groups were tested by analyses of variance, and differences between functional groups within habitats by means of Bonferroni *post hoc* tests. In addition, overall dispersal probabilities were calculated as the proportion of sampling stations receiving at least one seed from each of the different functional groups, with all habitats pooled. Similarly, for predation we used the probability of a single seed being consumed by rodents, and for browsing damage, the probability of an established sapling to be browsed. All analyses were performed with JMP 7.0 (SAS Inc.).

## Results and Discussion

Our results suggest that plant-animal interactions have a consistent influence on the functional components of the vegetation. By integrating field data on seed dispersal, seed predation, and browsing, we were able to infer how birds and mammals influence the recruitment and abundance of three functional groups in a Mediterranean landscape. Notably, seed-dispersing birds, as well as seed predators and browsers appear to favour the same type of vegetation across a degraded mosaic landscape.

**Figure 1:**
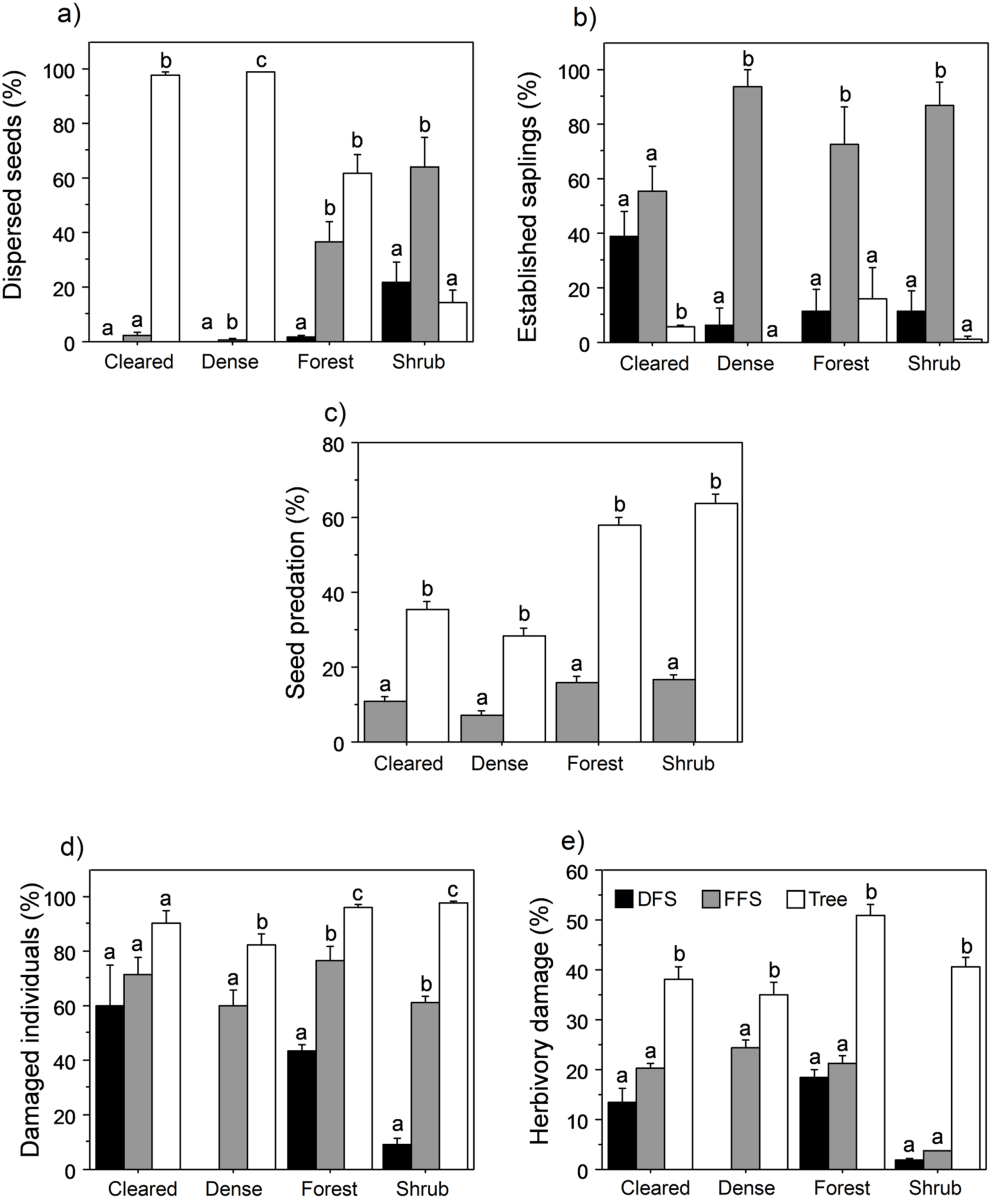
Intensity of the different biotic interactions among the different habitats (cleared and dense reforestation stands, native forest, and shrubland). A) Percentage of dispersed seeds from dry-fruited shrubs (DFS, black bars), fleshy-fruited shrubs (FFS, grey bars) and tree species (Tree, open bars). A total of 14,300 seeds were collected during the two study years: 5700 seeds of trees, 6000 of fleshy-fruited shrubs, and 2600 of dry-fruited shrubs (Mendoza et al. 2009). B). Percent of the established sapling bank belonging to the different functional groups among the different habitats. C) Percentage of depredated seeds in fleshy-fruited shrubs (FFS: *B. vulgaris* and *C. monogyna*; grey bars) and in tree species (*P. sylvestris*, *A. opalus*, and *S. aria*; open bars). D) Proportion of individuals damaged by browsers from the different functional groups among habitats. E) Percentage of buds damaged by browsers on attacked individuals of the different functional groups among habitats. Different letters denote differences at *P*<0.05 within habitats. Error bars represents +1SE.

By dispersing shrub species with higher recruitment probabilities than trees, birds become the first architects of these Mediterranean landscapes, which have a higher establishment probability than do tree species [25], [27]. Only 6.8% of the total seeds dispersed by birds were from zoochorus tree species, i. e. *T. baccata* and *S. aria*, the 98.8% of them were dispersed into native habitat (mean values for seeds of anemochorous trees dispersed into native habitat = 1023±280 seeds/ha; mean values for zoochorous trees = 85±84, F = 10.3, P = 0.03, *df* = 1) In fact, although most of the dispersed seeds came from tree species, mainly the anemochorous *P. sylvestris* (Fig. 1a, b; Table 1), the understory was dominated by fleshy-fruited shrubs (F = 101.5, *P*<0.0001; *df* = 2) irrespective of the habitat type (F = 0.7, *P* = 0.5; *df* = 3). Birds are important long-distance dispersers [33] and their movements may reduce differences in seed availability among landscape units [17], [20], [34], [35]. Thus, we found seeds of a wide diversity of zoochorous species in all habitats, even when parents were absent or scarce.

**Figure 2:**
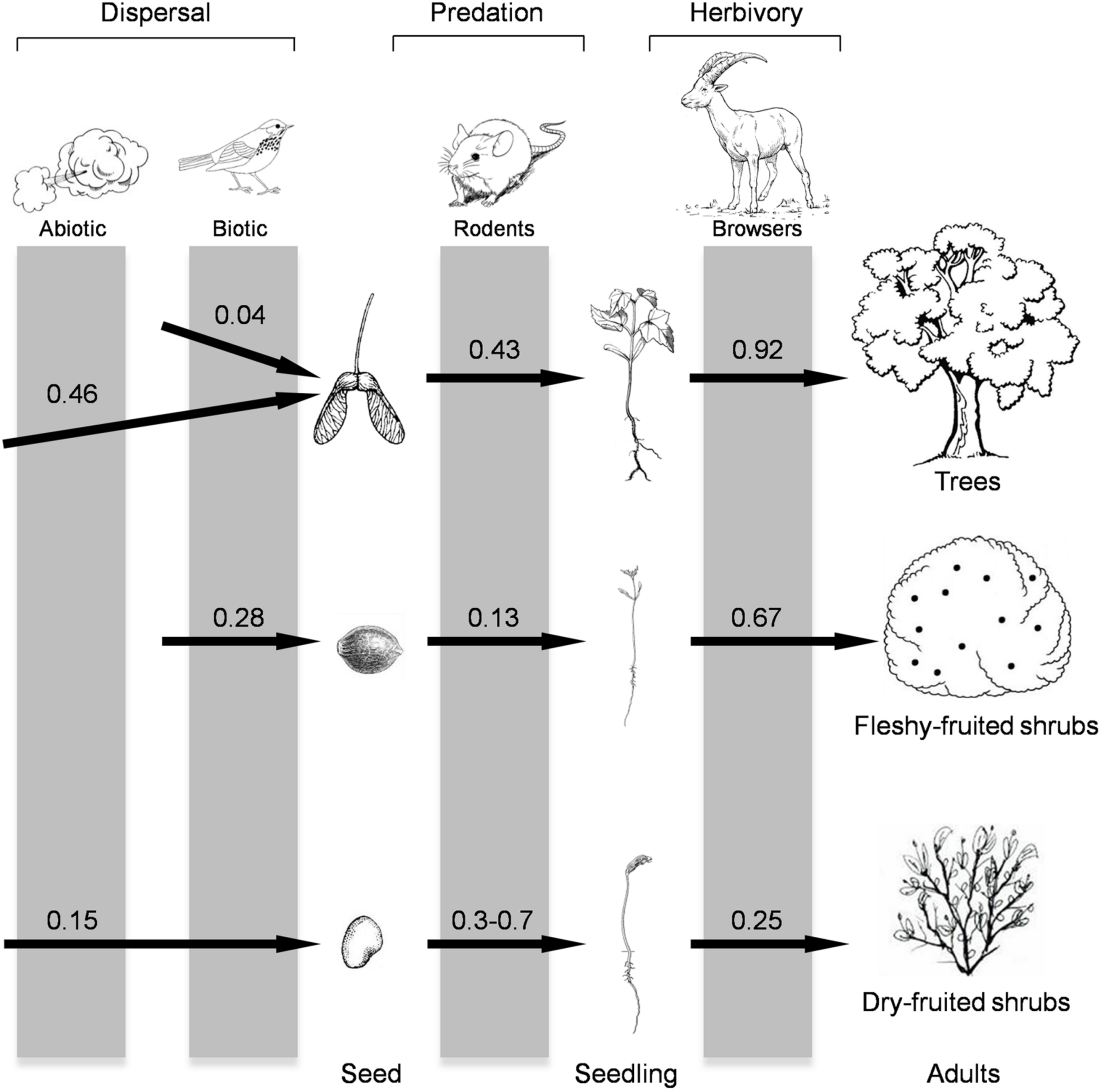
Probabilities (from 0 to 1) for the different plant functional groups (trees, fleshy-fruited shrubs and dry-fruited shrubs) of being affected by the different mutualistic and antagonistic ecological filters (dispersal, seed predation, and herbivory) among the different development phases. Dispersal data are the mean probability of a sampling station receiving at least one seed of the different groups; predation data are the probability of a single seed being consumed by rodents (dry-fruited shrubs range data based on the literature [36], [38]); and herbivory data represents the probability of an established individual (saplings in the case of trees) to be browsed. Data are pooled for the different habitats.

Although there were differences in the predation intensity among habitats (F = 50.6, *P*<0.0001; *df* = 3), seed predators preferentially consumed seeds from tree species (F = 493.2, *P*<0.0001; *df* = 1; Fig. 1c), whereas seeds from the dominant fleshy-fruited shrubs had a higher probability of escaping predation (Fig. 2). In the nearby oak forests of Sierra Nevada, acorn predation is even higher than for pine [30], [36], indicating that the two most abundant tree genera in Mediterranean mountains (*Pinus* and *Quercus*) undergo a high post-dispersal predation rate. Consequently, post-dispersal seed predation has the potential to filter the species pool available for recruitment of the woody community in a similar way in all landscape units, irrespective of the degree of degradation, reducing the number of propagules of dominant tree species and favouring further a shrub-like landscape, which is the type of landscape unit where rodents can find both food and refuge [26], [37]. Although we do not have data on seed predation in dry-fruited shrubs (mainly ballistic dispersal) in our study area, other studies indicate that their predation is much lower than for tree species, and are especially depredated by weevils before dispersal and by ants afterwards [38]–[41].

We found a strong browsing pressure (62% of individuals browsed and 21% damage on average for all functional groups and habitats) in the landscape mosaic of Sierra Nevada, similar to values found in other Mediterranean mountains or temperate woodlands [32], [42], [43]. However, this pressure did not affect all species in the sapling bank equally, since domestic and wild ungulates selectively browsed tree-species saplings far more intensely than the surrounding shrubs (F = 199.4, *P*<0.0001; *df* = 2; Fig. 1d, e). Besides the differences in herbivory pressure and intensity among habitats (F = 24.6, *P*<0.0001; *df* = 3), the same pattern of higer browsing over tree species was maintained. Previous studies in the same area [44], [45] have shown the same gradient of herbivore preferences for the same species, indicating the consistency of the reported pattern. This preference involves higher browsing damage for large numbers of tree saplings, which consequently underwent a reduction in annual height growth [32]. Furthermore, browsing in Mediterranean areas is more severe in summer when other food resources (herbaceous plants) for ungulates are less abundant, leaving less time for saplings to recover from the herbivory before winter [46]. All these factors exacerbate browsing damage, extending the time needed for saplings to reach maturity, and thereby lengthening the exposure time to browsers [20], [42].

Browsers therefore are able to constrain the successional trajectory in a native forest, changing the probabilities of transition from the recruitment pattern of the sapling bank to the adults of the canopy [31], [47], [48]. Furthermore, irrespective of the habitat type considered (whether native forest, pine plantations or shrubland), seed dispersal, seed predation, and browsing act synergistically to favour shrub recruitment and disfavour tree recruitment. In addition, we could expect the selective filtering process on woody vegetation imposed by mutualistic and antagonic animals to be enhanced by climatic conditions. As fleshy-fruited shrub species were the only ones that recruited in very dry years [25], our results imply that all landscape units, regardless of the degree of degradation, have a potential successional trend towards shrub dominance. This overall surge of shrubby species agrees with the shrub-encroachment trends described for many Mediterranean ecosystems [27], [49].

Our empirical results indicate that seed dispersers, seed predators, and browsers may act as a niche-based filter that contributes to a deterministic species assembly in this forest community, a process that may parallel effects of drought in aquatic mesocosm communities [50], or the effect of resource availability on forest recruitment [11]. These findings indicate for the first time that, although seed predation and browsing pressure differs quantitatively between adjacent landscape units, the selective filtering of the seed bank and saplings of woody species tends to be parallel among them. Furthermore, seedlings of fleshy-fruited shrubs are especially dominant in the understory of the two degraded landscape units: reforestation stands and shrubland [25], indicating a trend towards more abundance of fleshy-fruited shrubs in the overall mountain landscape. On the other hand, we should take into account that the dominance in shrub recruitment could also benefit, via plant-plant facilitation, the establishment of many other shrub and tree species [51]. Paradoxically, the expansion of fleshy-fruited shrubs does not lead inexorably to a total exclusion of tree species but also generates good niches for tree regeneration. As we have previously demonstrated, the presence of habitat-modifying pioneer shrubs may enhance species diversity by providing structural refuge to a broad array of woody shrubs and tree species which in turn would promote overall diversity of the woody community, especially in the most degraded landscape units [51].

In synthesis, our work identifies the major biotic processes that appear to be favoring a community dominated by shrubs versus trees because seed dispersers, predators, and herbivores together favored shrub dispersal and establishment versus trees among the different landscape units, irrespective of the degradation phase. The final outcome of this “animal-driven process” could be an overall increase in the functional similarity of the woody plant communities across the mosaic landscape. The concomitant proliferation of shrubs instead trees echoes a process already described in several neotropical fragmented forest landscapes (including Amazonia and the Atlantic Forest) comprising a case of widespread substitution of a wide range of native old-growth tree species (‘losers’), by native pioneer (‘winners’) [24]. In addition, this type of “novel” shrub-dominated community is better adapted to cope with the expected changes in climate for the coming decades in this area under a climate-change scenario [27], [52], because shrubs are less vulnerable to drought and browsing than are tree species. Over the long term, biotic as well as abiotic factors may converge to promote increased dominance of the most drought-tolerant species, creating a favourable selective environment for woody plants with a similar combination of functional traits (zoochorous, unpalatable, drought resistant) and thus enhancing shrubland expansion in the overall mountain landscape, irrespective of the degree of past human management.
